# Clinicopathological factors associated with sentinel lymph node detection in non-small-cell lung cancer

**DOI:** 10.1186/s13019-024-02632-y

**Published:** 2024-03-19

**Authors:** Christophe Wollbrett, Joseph Seitlinger, Florent Stasiak, Juliette Piccoli, Arthur Streit, Joelle Siat, Guillaume Gauchotte, Stéphane Renaud

**Affiliations:** 1 Department of Thoracic Surgery, Nancy Regional University Hospital, Vandoeuvre-lès- Nancy, 54500 France; 2 Department of Pathology and Molecular Biology, Nancy Regional University Hospital, Vandoeuvre-lès-Nancy, 54500 France; 3 Research Unit INSERM U1256, NGERE Unit, Lorraine University, Vandoeuvre-lès-Nancy, 54500 France

**Keywords:** Sentinel lymph node, Near-infrared fluorescence, Indocyanine green, Electromagnetic navigation bronchoscopy, Non-small-cell lung cancer

## Abstract

**Background:**

Mapping of the pulmonary lymphatic system by near-infrared (NIR) fluorescence imaging might not always identify the first lymph node relay. The aim of this study was to determine the clinicopathologic factors allowing the identification of sentinel lymph nodes (SLNs) by NIR fluorescence imaging in thoracic surgery for non-small-cell lung cancer (NSCLC).

**Methods:**

We conducted a retrospective review of 92 patients treated for suspected or confirmed cN0 lung cancer with curative intent who underwent an intraoperative injection of indocyanine green (ICG) either by direct peritumoral injection or by endobronchial injection using electromagnetic navigational bronchoscopy (ENB). After exclusion of patients for technical failure, benign disease and metastasis, we analyzed the clinicopathologic findings of 65 patients treated for localized-stage NSCLC, comparing the group with identification of SLNs (SLN-positive group) with the group without identification of SLNs (SLN-negative group).

**Results:**

Forty-eight patients (73.8%) were SLN-positive. Patients with SLN positivity were more frequently female (50%) than the SLN-negative patients were (11.8%) (*p* = 0.006). The mean value of diffusing capacity for carbon monoxide (DLCO) was lower among the patients in the SLN-negative group (64.7% ± 16.7%) than the SLN-positive group (77.6% ± 17.2%, *p* < 0.01). The ratio of forced expiratory volume in one second to forced vital capacity (FEV1/FCV) was higher in the SLN-positive group (69.0% vs. 60.8%, *p* = 0.02). Patients who were SLN-negative were characterized by a severe degree of emphysema (*p* = 0.003). There was no significant difference in pathologic characteristics. On univariate analyses, age, female sex, DLCO, FEV1/FVC, degree of emphysema, and tumor size were significantly associated with SLN detection. On multivariate analysis, DLCO > 75% (HR = 4.92, 95% CI: 1.27–24.7; *p* = 0.03) and female sex (HR = 5.55, 95% CI: 1.25–39.33; *p* = 0.04) were independently associated with SLN detection.

**Conclusions:**

At a time of resurgence in the use of the sentinel lymph node mapping technique in the field of thoracic surgery, this study enabled us to identify, using multivariate analysis, two predictive factors for success: DLCO > 75% and female sex. Larger datasets are needed to confirm our results.

**Supplementary Information:**

The online version contains supplementary material available at 10.1186/s13019-024-02632-y.

## Background

Sentinel lymph node (SLN) mapping consists of studying the first lymph node relay, identified after injection of a tracer around a tumor [1]. This tracer migrates into the lymphatic network and allows the identification of the first lymph node draining the tumor. An absence of lymph node metastasis in this SLN indicates a very low risk of lymph node metastasis to later nodes [2]. An equally important potential role of SLN mapping may be directing pathologic assessment to specific sentinel nodes and applying more sensitive techniques to a limited amount of tissue to detect occult micrometastatic disease [3].

Some authors have proposed using SLN mapping for non-small-cell lung cancer (NSCLC) to optimize selective dissection, which they advocate by drawing a parallel with breast cancer and melanoma, where guidelines are well codified with good results [4, 5]. In NSCLC, this technique has been studied but has not been widely disseminated due to the rather low SLN identification rate [6–13]. In this context, complete mediastinal lymphadenectomy remains the standard of care in lung cancer surgery [14].

The Cancer and Leukemia Group B multicenter prospective phase II trial of SLN mapping in stages I and II NSCLC using Technetium-99 showed a low sensitivity of the technique (51%) and achieved a 61.5% SLN identification rate [15]. Since then, other tracers, radiopaque and isotopic, as well as other means of detection, have been tested, with variable success [16]. The technique that seems the most promising is injecting indocyanine green (ICG) endobronchially, as reported by Phillips et al. [13]. These authors demonstrated a significantly higher rate of SLN identification with a total ICG injection dose ≥ 1 mg, albumin dissolvent, and lung ventilation after injection.

Based on their method, we recently conducted a study to assess the safety and feasibility of an intraoperative near-infrared (NIR)–guided SLN approach to lymphatic mapping in patients with lung cancer. We found that NIR lymphatic mapping was feasible, safe and good at identifying regional lymph nodes [17, 18]. Why SLN detection fails in some patients has not yet been evaluated. Therefore, we aimed to assess factors interfering with SLN detection.

The aim of this study was to evaluate clinicopathologic factors related to NIR fluorescence–guided SLN identification in thoracic surgery.

## Methods

### Patients

We retrospectively reviewed 92 consecutive patients from a prospective database who underwent major lung resection (i.e., lobectomy and segmentectomy) for confirmed or suspected primary lung cancer at Nancy Regional University Hospital (Vandoeuvre-lès-Nancy, France) between December 2020 and March 2023 and who underwent mapping of the pulmonary lymphatic system by NIR fluorescence imaging. We focused on clinical (c) stages I to IIA according to the eighth edition of the Tumor-Node‐Metastasis Classification of the Union for International Cancer Control [19]. Those with resection of benign tumors, small-cell lung cancer, metastatic diseases or technical failure (i.e., intrapleural injection, fluorescence column malfunction) were excluded.

**Fig. 1:**
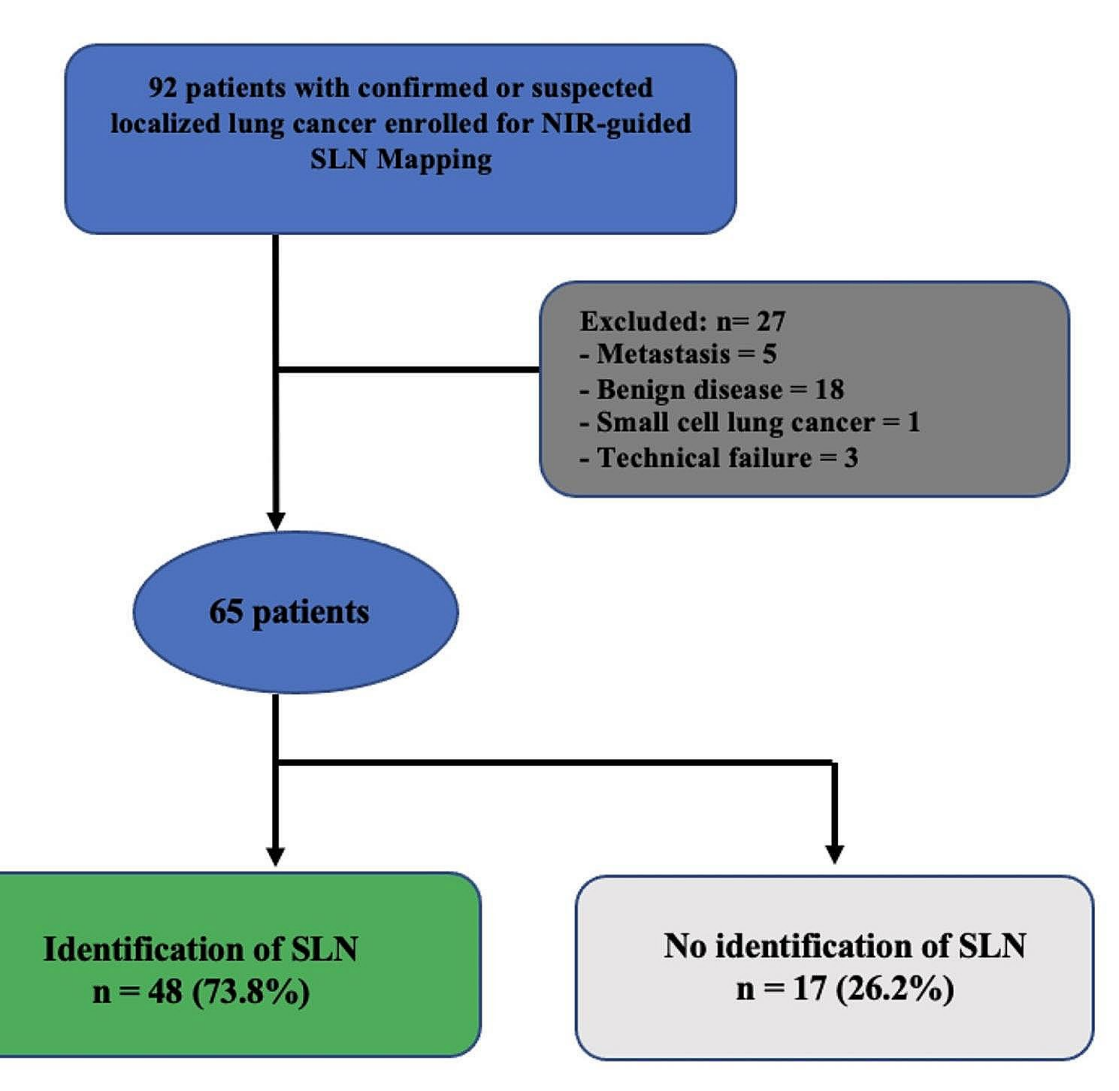
Flowchart of patient selection. NIR, Near-infrared; SLN, sentinel lymph node

Patients were divided into two groups: patients with identification of SLNs (SLN-positive group) and patients without identification of SLNs (SLN-negative group). A flowchart of the enrolled patients is shown in Fig. 1.

Patient data and clinicopathologic characteristics of resected tumors as well as the results of positron emission tomography (PET) and chest computed tomography (CT) in the two groups were compared.

Each operation was either open surgery, video-assisted thoracoscopic surgery (VATS) or robot-assisted thoracoscopic surgery (RATS), according to surgeon preference. Radical lymph node dissection was routinely performed as recommended by ESTS guidelines [14]. None of the patients underwent neoadjuvant therapy.

This project was approved by our institutional review board.

### Intraoperative technique

The technique of peritumoral ICG injection, either transpleural or transbronchial, mediated by electromagnetic navigation bronchoscopy and NIR imaging, was performed as previously described [17]. In summary, 1 mL of ICG was injected peritumorally, and the assessment of the SLN with an NIR camera (Visionsense©, Medtronic, Minneapolis, USA) was initiated after at least 5 min of bipulmonary ventilation. In all cases, if an SLN was fluorescent, it was resected, and a systematic lymph node dissection was performed.

**Fig. 2:**
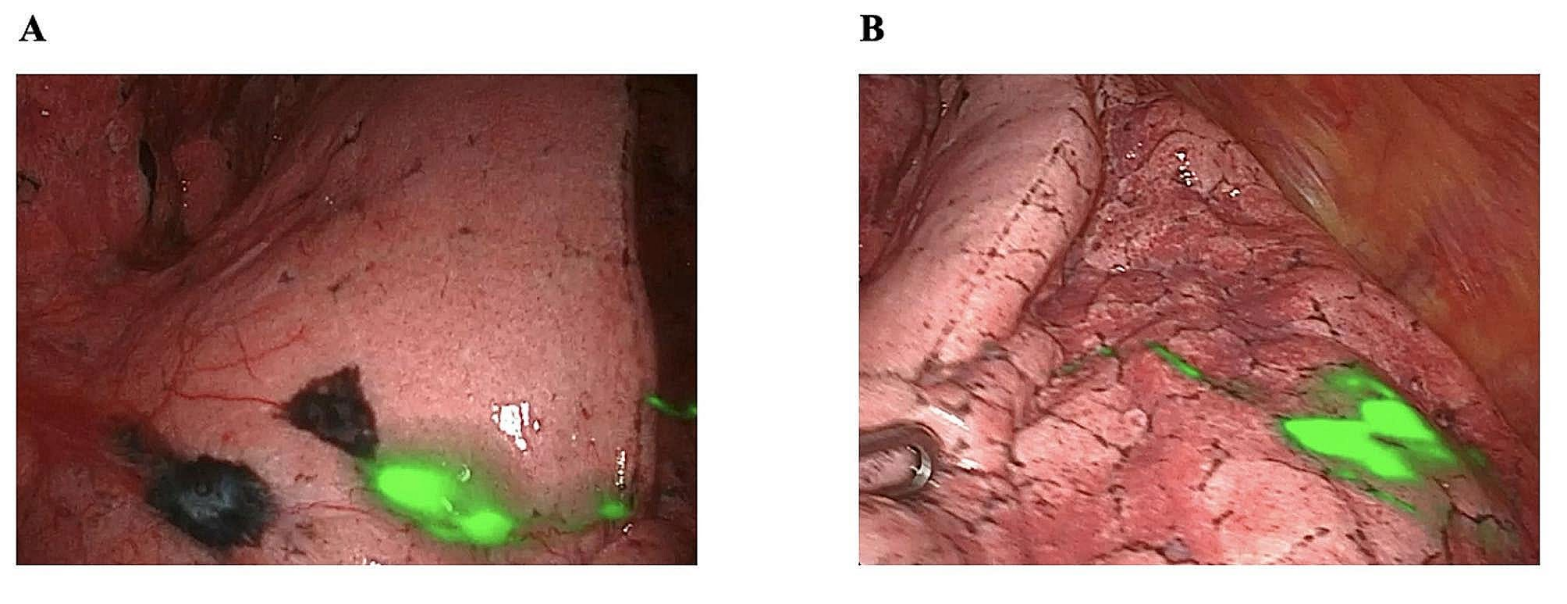
NIR-Guided sentinel lymph node. **A**: The thoracoscopic ICG fluorescence imaging system showed sentinel nodes at the peripheral lymph node. **B**: The lymphatic pathway is clearly seen with NIR on the surface of the lung, starting from the lung nodule located in the left lower lobe

A lymph node (LN) was considered to be an SLN if it was identified by at least one of the following criteria: [1] the LN was fluorescent or [2] the LN had a fluorescent-stained afferent lymphatic vessel leading to it (Fig. 2).

Three surgeons, two seniors and one resident, participated in this trial and enrolled between 6 and 45 patients each.

### 18 F-FDG PET/CT scanning and image analysis

Since PET has been described as a predictive tool for SLN identification [13], we included PET data. The [18]F-fluorodeoxyglucose positron emission tomography results were reviewed by two attending nuclear medicine physicians. The PET image parameter we used was the mean maximum standardized uptake value (SUVmax) of the lesion. The cutoff value of SUVmax was set to 3.5 based on a previous study [13].

Visual emphysema was defined as disrupted lung vasculature and parenchyma with low attenuation occupying any lung zone (at least trace) on chest CT, as evaluated by radiologists using the National Emphysema Treatment Trial or Fleischner Society guidelines [20, 21]. Based on these guidelines, specific percentages of visual were used to assess emphysematous lung tissue destruction at CT (i.e., mild: 0 – 25%; moderate: 26 – 50%; and severe: ≥ 51%).

Subsolid and solid nodules were distinguished according to the presence of ground-glass opacity (GGO). In the subsolid nodules, a ground glass nodule was defined as a nodule without a solid component, and part-solid nodules were defined as lung lesions with both a GGO and solid component, according to guidelines from the Fleischner Society in 2017 [22].

### Histologic evaluation

For each patient, hematoxylin-, eosin- and saffron-stained tissue slides were analyzed by pathological specialists. On each slide, the following items were analyzed: predominant architecture, presence of spread through air spaces (STAS), visceral pleural invasion (VPI), visceral pleural distance, perineural neoplastic invasion, vascular invasion, tumor size and tumor location. VPI was considered to be positive when tumor cells extended beyond the elastic layer of the pleura, as determined by elastic staining. Pleural (PL) invasion was classified into the following: PL0, tumor with no pleural involvement beyond the elastic layer; PL1, tumor invading beyond the elastic layer of the visceral pleura but not exposed on the pleural surface; PL2, tumor invading the pleural surface. The clinical and pathological stages were reassessed according to the 8th edition of the tumor-node‐metastasis classification of the Union for International Cancer Control [19].

Immunohistochemistry was performed on deparaffinized and rehydrated tissue sections. The percentage of tumor cells stained with an anti-programmed death-ligand 1 (PD-L1) antibody was determined by evaluating tumor cells showing linear membrane staining (circumferential or partial) of any intensity. PD-L1 status was considered negative if the percentage of labeled cells was < 1%, positive if the rate was ≥ 1%, and strongly positive if the rate was ≥ 50% [23].

In 38 and 19 patients, epidermal growth factor receptor (*EGFR*) and V-Ki-ras2 Kirsten rat sarcoma viral oncogene homolog (*KRAS*) gene mutations were performed by previously described methods [24].

### Statistical analysis

Patient data and clinicopathologic characteristics are presented as mean (standard deviation, SD) or median (interquartile range, IQR) for continuous variables and as number (percentage) for categorical variables. Student’s T test for continuous variables and the chi-squared test for categorical variables were used to test for differences. All tests were two-tailed, and a p value less than 0.05 was considered statistically significant.

The multivariable analysis was performed using stepwise logistic regression, and the model-building process was a backward variable selection approach with a significance cutoff of < 0.2 to minimize the Akaike information criterion (AIC) [25].

We examined the cutoff value of the diffusing capacity for carbon monoxide (DLCO), the forced expiratory volume in one second/forced vital capacity ratio (FEV1/FVC) and the forced expiratory volume in one second (FEV1) using receiver operating characteristic (ROC) curves, which yielded the highest combined sensitivity and specificity for distinguishing SLN.

All analyses were performed using R (version 4.2.2).

## Results

### Clinical characteristics associated with sentinel lymph node detection

According to our inclusion criteria, among the 92 patients eligible, sixty-five patients with primary NSCLC were retrospectively reviewed. Table 1 shows the clinical characteristics of the 17 (26.2%) SLN-negative patients and 48 (73.8%) SLN-positive patients.

**Table 1:** Clinical characteristics associated with sentinel lymph node detection

	SLN-negative group (*n* = 17)	SLN-positive group (*n* = 48)	p
Median age (years)	70 (IQR 65–77)	65 (IQR 61–72)	0.06
Female sex	2 (11.8)	24 (50)	**0.006**
Smoking use	15 (88.2)	36 (75.0)	0.25
DLCO (%)	64.7 ± 16.7	77.6 ± 17.2	**< 0.01**
FEV1/FVC (%)	60.8 ± 14.5	69.0 ± 11.7	**0.02**
FEV1 (%)	77.3 ± 20.9	90.6 ± 20.2	**0.02**
Lobe Right upper Right middle Right lower Left upper Left lower	3 (17.6) 2 (11.8) 5 (29.4) 5 (29.4) 2 (11.8)	17 (35.4) 2 (4.2) 11 (22.9) 16 (33.3) 2 (4.2)	0.42
Nodule density Solid Subsolid	15 (88.2) 2 (11.8)	37 (77.1) 11 (22.9)	0.32
Degree of emphysema Light-moderate Severe	8 (47.1) 9 (52.9)	40 (83.3) 8 (16.7)	**0.003**
Extent of operation Segmentectomy Lobectomy	10 (58.8) 7 (41.2)	22 (45.8) 26 (54.2)	0.36
Surgical approach VATS RATS Open thoracotomy	13 (76.5) 4 (23.5) 0 (0)	40 (83.3) 3 (6.3) 5 (10.4)	0.07
Marking Method Transpleural ENB	6 (35.3) 11 (64.7)	8 (16.7) 40 (83.3)	0.11
SUVmax SUVmax < 3.5 SUVmax ≥ 3.5	7 (41.2) 10 (58.8)	20 (41.7) 28 (58.3)	0.72

Values are n (%) or mean (SD) unless otherwise indicated

DLCO, diffusing capacity for carbon monoxide; ENB, electromagnetic navigation bronchoscopy; FEV1, forced expiratory volume in one second; FEV1/FVC, forced expiratory volume in one second to forced vital capacity ratio; IQR, interquartile range; RATS, robotic-assisted thoracoscopic surgery; SD, standard deviation; SLN, sentinel lymph node; SUVmax, maximum standardized uptake value; VATS, video-assisted thoracoscopic surgery

Patients with SLN positivity were more frequently female (50% vs. 11.8%, *p* = 0.006). The mean value of DLCO was 64.7% ± 16.7% among the patients with unidentifiable SLN, which was significantly lower than the 77.6% ± 17.2% among the patients with identifiable SLN (*p* < 0.01). FEV1/FCV was significantly higher in the SLN-positive group (69.0% vs. 60.8%, *p* = 0.02). The mean value of FEV1 was 77.3% ± 20.9% among the patients with unidentifiable SLN, which was significantly lower than the 90.6% ± 20.2% among the patients with identifiable SLN (*p* = 0.02). Patients who were SLN-negative were characterized by significantly more severe emphysema (*p* = 0.003).

### Pathologic characteristics associated with sentinel lymph node detection

As shown in Table 2, there were no significant differences in pathologic factors between groups.

**Table 2:** Pathologic characteristics associated with sentinel lymph node detection

	SLN-negative group (*n* = 17)	SLN-positive group (*n* = 48)	p
Tumor size (cm) ≤ 1 cm 1.1 − 2 cm 2.1 − 3 cm ≥ 3.1 cm	1 (5.9) 7 (41.2) 7 (41.2) 2 (11.8)	12 (25.0) 22 (45.8) 8 (16.7) 6 (12.5)	0.13
Location Central Peripheral	2 (11.8) 15 (88.2)	5 (10.4) 43 (89.6)	0.88
Histology Adenocarcinoma Squamous cell carcinoma Others	8 (47.1) 7 (41.2) 2 (11.8)	36 (75.0) 8 (16.7) 4 (8.3)	0.09
T stage T1a T1b T1c T2a T2b	1 (5.9) 5 (29.4) 6 (35.3) 4 (23.5) 1 (5.9)	12 (25.0) 19 (39.6) 7 (14.6) 10 (29.8) 0 (0)	0.14
N stage N1 N2	1 (5.9) 0 (0)	3 (6.3) 2 (4.2)	0.44
Stage IA IB IIA IIB IIIA	11 (64.7) 4 (23.5) 1 (5.9) 1 (5.9) 0 (0)	36 (75.0) 7 (14.6) 1 (2.1) 2 (4.2) 2 (4.2)	0.74
Visceral pleural invasion PL0 + PL1 PL2	2 (11.8) 0 (0)	4 (8.3) 1 (2.1)	0.88
Visceral pleural distance (cm)	0.55 ± 0.73	0.45 ± 0.57	0.5
STAS	9 (52.9)	19 (39.6)	0.34
Perineural neoplastic invasion	1 (5.9)	1 (2.1)	0.44
Vascular invasion	4 (23.5)	10 (20.8)	0.82
*EGFR* mutation	0 (0)	4 (8.3)	0.16
PD-L1 status PD-L1 < 1% PD-L1 1 − 49% PD-L1 ≥ 50%	8 (47.1) 5 (29.4) 4 (23.5)	28 (63.6) 12 (27.3) 4 (9.1)	0.245
*KRAS* mutation	5 (29.4)	8 (16.7)	0.45

Values are n (%) or mean (SD) unless otherwise indicated

cm, centimeter; *EGFR*, epidermal growth factor receptor; *KRAS*, V-Ki-ras2 Kirsten rat sarcoma viral oncogene homolog; PD-L1, Programmed death-ligand 1; PL, Pleural; SD, standard deviation; SLN, sentinel lymph node; STAS, Spread through air spaces

The mean tumor size was not significantly different between the two groups (2.0 cm in the SLN-negative group versus 1.7 cm in the SLN-positive group, *p* = 0.23), nor was the distribution of central and peripheral locations (*p* = 0.88).

The incidences of VPI, perineural neoplastic invasion, STAS and vascular invasion were not significantly different between the two groups.

There was no significant difference in *EGFR* mutational status (*p* = 0.16), *KRAS* mutations (*p* = 0.45) or PD-L1 status (*p* = 0.245) between the two groups. Since *KRAS* and *EGFR* mutational status were examined in only 29.5% (13/44) and 9.1% (4/44) of the patients with adenocarcinoma enrolled in this study, respectively, they were not included as study variables in logistic regression.

### Univariate and multivariate analyses of SLN detection

ROC curves for DLCO, FEV1/FVC and FEV1 were established to determine the optimal cutoff value for predicting SLN detection. For DLCO, the area under the ROC curve (AUC) was 0.69, and 75% was calculated as the best cutoff value (sensitivity 60.4%, specificity 81.3%). For FEV1/FVC, the AUC was 0.687, and 70% was the cutoff value (sensitivity 74.5%, specificity 64.7%). For FEV1, the AUC was 0.67, and 93% was calculated as the best cutoff value (sensitivity 70.3%, specificity 63.4%).

Univariate analysis (Table 3) showed a significantly higher detection rate in female patients (HR = 7.5, 95% CI: 1.85–50.95; *p* = 0.01), in patients with high DLCO compared to low DLCO (HR = 6.61, 95% CI: 1.84–31.73; *p* = 0.01), in patients with high FEV1/FVC compared to low FEV1/FVC (HR = 2.73, 95% CI: 0.86–9.71; *p* = 0.1), and in patients with high FEV1 compared to low FEV1 (HR = 3.09, 95% CI: 0.98–10.98; *p* = 0.06) and a lower detection rate in older patients (HR = 0.93, 95% CI: 0.86–1; *p* = 0.06). Severe emphysema (HR = 0.18, 95% CI: 0.05–0.59; *p* = 0.005) and tumor size between 2.1 and 3 cm (HR = 0.1, 95% CI: 0–0.68; *p* = 0.04) were significantly associated with less SLN detection.

**Table 3:** Univariate analysis of factors associated with SLN identification by logistic regression

	HR [95% CI]	p
Female sex	7.5 [1.85–50.95]	**0.01**
Age	0.93 [0.86–1]	**0.06**
Smoking	0.4 [0.06–1.71]	0.27
DLCO (%)		
Low (≤ 75)	Ref	**-**
High (> 75)	6.61 [1.84–31.73]	**0.01**
FEV1/FVC (%)		
Low (≤ 70)	Ref	**-**
High (> 70)	2.73 [0.86–9.71]	**0.1**
FEV1 (%)		
Low (≤ 93)	Ref	**-**
High (> 93)	3.09 [0.98–10.98]	**0.06**
Degree of emphysema		
Light-moderate	Ref	-
Severe	0.18 [0.05–0.59]	**0.005**
Extent of operation		
Lobectomy	Ref	-
Segmentectomy	0.59 [0.19–1.8]	0.36
Nodule density		
Subsolid	Ref	-
Solid	0.45 [0.06–1.94]	0.33
Tumor size (cm)		
≤ 1 cm	Ref	
1.1 − 2 cm	0.26 [0.01–1.73]	0.23
2.1 − 3 cm	0.1 [0–0.68]	**0.04**
≥ 3.1 cm	0.25 [0.01–3.12]	0.29
N+	1.86 [0.27–37.1]	0.58
Visceral pleural invasion	0.87 [0.17–6.53]	0.88
Vascular invasion	0.86 [0.24–3.54]	0.82

cm, centimeters; DLCO, diffusing capacity for carbon monoxide; FEV1, forced expiratory volume in one second; FEV1/FVC, forced expiratory volume in one second to forced vital capacity ratio; HR, hazard ratio; 95% CI, confidence interval at 95%; SLN, sentinel lymph node

Multivariate analysis with an AIC value of 60 enabled us to identify three variables to input into our multivariable model (Table 4). Two variables were associated with SLN detection: DLCO > 75% (HR = 4.92, 95% CI: 1.27–24.7; *p* = 0.03) and female sex (HR = 5.55, 95% CI: 1.25–39.33; *p* = 0.04). The results of the multivariable models and their corresponding AICs are shown in the Additional file 1.

**Table 4:** Multivariate analysis of factors associated with SLN identification by logistic regression

	HR [IC 95%]	p
Age	0.94 [0.86–1.03]	0.2
Female sex	5.55 [1.25–39.33]	**0.04**
DLCO (%)		
Low (≤ 75)	Ref	-
High (> 75)	4.92 [1.27–24.7]	**0.03**

DLCO, diffusing capacity for carbon monoxide; HR, hazard ratio; 95% CI, confidence interval; SLN, sentinel lymph node

## Discussion

In this study, we identified two clinical-demographic factors, namely, DLCO above 75% and female sex, to be associated with successful SLN mapping. On the other hand, factors related to lung cancer itself, including tumor histology, grade and molecular characteristics, were not found to be associated with successful mapping.

SLN mapping has become standard in the surgical management of patients with breast cancer and melanoma [1, 26]. Most recently, the assessment of SLN in the field of lung surgery by using NIR fluorescence imaging has appeared promising. In a recent study, our team concluded that SLN mapping could be helpful for identifying occult micrometastases and improving staging [17]. By guiding lymph node dissection, SLN mapping could help reduce postoperative morbidity, which is rare but can occur due to hemorrhage, recurrent nerve paralysis, esophageal wounds, chylothorax, worsening of bronchial congestion by pulmonary denervation, and bronchial fistula by devascularization of the stump [27]. Moreover, as we have found [18], station 11 LN is not always the first relay. Hence, SLN mapping could be helpful in the era of segmentectomy in the future. Indeed, it might help to identify the real first LN relay, allowing a more accurate intraoperative pN0 confirmation by frozen section, which is mandatory in case of segmentectomy. Finally, in the era of immunotherapy, systematic lymph node dissection in patients with an uninvolved SLN (confirmed by frozen analysis) should not be automatic. Indeed, previous authors have reported a better response to immune checkpoint blockade in patients with absence of complete LN dissection in comparison with a systematic treatment [28]. For all these reasons, to optimize the SLN mapping technique in NSCLC patients, it is critical to understand factors impacting the success of SLN identification.

A literature review regarding factors associated with SLN detection revealed that most experience with this technique comes from the treatment of breast cancer [29–31], though some SLN detection factors in thoracic surgery have been described in the literature. Nomori et al. [12] and Yashimata et al. [32] have previously described patients with chronic obstructive pulmonary disease (COPD), low FEV1/FVC ratio and lung emphysema as predictive factors of SLN identification. In our study, in accordance with those findings, patients with DLCO < 75% and FEV1/FVC < 70% ratio and those who have a severe degree of emphysema have significantly more SLN detection failure (although in multivariate analysis, only low DLCO was statistically significant). We hypothesize that lung tissue in patients with low DLCO could have a lymphatic dysfunction, resulting in a lower SLN identification rate. A recent study carried out on mouse model exposed to cigarette smoke has pointed an increased number of lymphatic vessels in peripheral lung compartments but associated to lymphatic dysfunction with impaired drainage, decreased leukocyte trafficking, and prothrombotic lymph resulting in lymphatic thrombosis [33]. We concurrently observed that the rate of SLN identification was marginally lower in older patients. In breast cancer, it has been reported that the SLN identification rate is significantly lower in older patients [31]. This can be partially explained by the progressive infiltration of parenchymal lymph node with fat cells in older patients [34]. We can therefore hypothesize that these discriminating changes in the peritumor environment of NSCLC could be decisive and could significantly reduce the probability of SLN detection.

More notably, we reported a high incidence of SLN detection in females. To the best of our knowledge, this is the first investigation to find an association between SLN detection and sex. Indeed, only 11.8% of SLN-negative patients were women, whereas half of the SLN-positive patients were women. Our multivariate analysis is additional evidence that female sex seems to be an independent predictor of successful SLN mapping. The hazard ratio of SLN-positive identification for female patients was more than 5. This might be explained by a hormonal theory, in which the process of lymphangiogenesis is partly initiated by the activation of estrogen receptor α (ERα), located on the plasma membrane of the endothelial cells of the lymphatic vessels [35]. Moreover, in a study carried out on murine models, the authors identified a protective action of 17β-estradiol targeting ER α on the lymphatic endothelium [36]. However, the hormonal control of the lymphatic system remains largely unexplored, so additional translational research is warranted to understand the predictive value of female sex in SLN detection.

A recent study showed that radiologically solid nodules (compared to subsolid nodules) and anatomic resection (compared to wedge resection) significantly increased the likelihood of SLN identification [13]. These observations might be partially explained by the fact that wedge resection leads to a less extensive dissection of vascular and bronchial structures, probably leading to an underexploration of LNs. On the other hand, in our work, we did not observe any statistically significant difference in terms of nodule density or extent of operation. However, our cohort had a high percentage of patients with solid nodules (80% with solid nodules), and we retrospectively reviewed patients who underwent major lung resection (i.e., lobectomy and segmentectomy).

The question arises whether tumor and molecular characteristics are important for SLN mapping in NSCLC. To date, only one study has reported data on the use of NIR image–guided SLN mapping in NSCLC and concluded that there were no significant differences in patient characteristics between the SLN and non-SLN groups, including histologic subtype or grade [37]. In our study, we observed no significant association between tumor pathological features and pulmonary lymphatic mapping. Interestingly, previous studies have shown that *KRAS* codon 12 mutation is associated with a significant increase in the production of vascular endothelial growth factor (VEGF), which is involved in lymphangiogenesis [38]. However, the lack of data subdivided by molecular alterations in our study prevents us from reaching a definitive conclusion. Studies on the mapping of the pulmonary lymphatic system according to their mutational status seem urgent, as this hypothesis merits further investigation.

With the advent of immunotherapy in the therapeutic landscape of localized NSCLC [39], one question remains unanswered: Will SLN mapping after neoadjuvant immunochemotherapy treatment have the same rate of success as SLN mapping not done after it? Indeed, it is widely accepted by most surgical teams that resection after induction immunochemotherapy can sometimes be more challenging due to a vigorous reaction around the lymph nodes. This observation needs to be investigated in samples treated with a neoadjuvant regimen.

Our study has some limitations that should be taken into account when interpreting the results. It is a retrospective cohort study based on a relatively small sample size and conducted at a single institution. However, data on sentinel lymph node in lung cancer are based so far on small cohort in the literature, with data arising from only few centers in the world. So far, this study is the largest cohort on SLN mapping in lung, with the only one focusing on predictive factors of success. This is a preliminary study, providing basis for larger international multi-center studies. Otherwise, our study covers a 3-year period during which three surgeons with different levels of experience performed the procedures. Differences in experience may also have influenced the SLN detection rate. Indeed, Liptay et al. [15] and Yashimata et al. [32] reported a link between SLN detection and surgeon experience. However, ours is the largest reported cohort of patients undergoing mapping of the pulmonary lymphatic system by NIR fluorescence imaging in NSCLC in whom the relationship between clinicopathological characteristics and identification of SLNs has been tested. Larger clinicopathological studies are required to overcome these limitations.

## Conclusions

In summary, female sex and a DLCO greater than 75% emerged as independent predictors of SLN identification in thoracic surgery by NIR fluorescence imaging in NSCLC. This study shows the importance of clinical factors compared to pathological factors, and these factors could explain some of our detection failures. Nevertheless, further large-scale prospective studies are needed to answer these questions, particularly in neoadjuvant immunotherapy settings.

### Electronic supplementary material

Below is the link to the electronic supplementary material.

Supplementary Material 1

## Data Availability

The datasets used and/or analysed during the current study are available from the corresponding author on reasonable request.

## Abbreviations

AIC: Akaike information criterion
AUC: area under the ROC curve
COPD: chronic obstructive pulmonary disease
CT: computed tomography.
DLCO: diffusing capacity for carbon monoxide
EGFR: epidermal growth factor receptor
ENB: electromagnetic navigational bronchoscopy
Er: #x03B1; estrogen receptor α
ESTS: European Society of Thoracic Surgeons
FEV1: forced expiratory volume in one second
FEV1: FCV forced expiratory volume in one second to forced vital capacity
GGO: ground-glass opacity
HR: hazard ratio
ICG: indocyanine green
IQR: interquartile range
KRAS: V-Ki-ras2 Kirsten rat sarcoma viral oncogene homolog
LN: lymph node
NIR: near-infrared
NSCLC: non-small-cell lung cancer
PD: L1 anti-programmed death-ligand 1
PET: positron emission tomography
PL: pleural
RATS: robot-assisted thoracoscopic surgery
ROC: receiver operating characteristic
SD: standard deviation
SLNs: sentinel lymph nodes
STAS: spread through air spaces
SUVmax: standardized uptake value
VATS: video-assisted thoracoscopic surgery
VEGF: vascular endothelial growth factor
VPI: visceral pleural invasion
95% CI: confidence interval at 95%
