# Supplementary material for: Clinicopathological factors associated with sentinel lymph node detection in non-small-cell lung cancer
Source: J Cardiothorac Surg. 2024 Mar 19;19:145. doi: 10.1186/s13019-024-02632-y (PMC10949663; doi:10.1186/s13019-024-02632-y)
Supplement: Supplementary file 1 — Supplementary Material 1 [file 13019_2024_2632_MOESM1_ESM.docx]

**Additional file 1** Multivariate models and corresponding AICs.

## **Model 1**

|  | **HR [IC 95%]** | ***p*** |
| --- | --- | --- |
| Age | 0.94 [0.86–1.03] | 0.20 |
| Female sex | 6.05 [1.4–42.43] | **0.03** |
| Tumor size (cm) |  |  |
| ≤ 1 cm | Ref | - |
| 1.1 cm - 2 cm | 0.41 [0.02–3.16] | 0.45 |
| 2.1 cm - 3 cm | 0.18 [0.01–1.6] | 0.17 |
| ≥ 3.1 cm | 0.55 [0.02–8.75] | 0.68 |

**AIC = 72**

## **Model 2**

|  | **HR [IC 95%]** | ***p*** |
| --- | --- | --- |
| Age | 0.95 [0.85–1.05] | 0.32 |
| Female sex | 11.13 [1.77–220.13] | **0.03** |
| DLCO (%) |  |  |
| Low (≤ 75) | Ref | - |
| High (> 75) | 5.67 [1.35–31.3] | **0.03** |
| Tumor size (cm) |  |  |
| ≤ 1 cm | Ref | - |
| 1.1 cm - 2 cm | 0.36 [0.02–3.25] | 0.41 |
| 2.1 cm - 3 cm | 0.25 [0.01–2.67] | 0.29 |
| ≥ 3.1 cm | 0.74 [0.02–15.95] | 0.85 |

**AIC = 65**

## **Model 3**

|  | **HR [IC 95%]** | ***p*** |
| --- | --- | --- |
| Age | 0.94 [0.86–1.04] | 0.2 |
| Female sex | 5.55 [1.25–39.33] | **0.04** |
| DLCO (%) |  |  |
| Low (≤ 75) | Ref | - |
| High (> 75) | 4.92 [1.27–24.7] | **0.03** |

**AIC = 60**

## **Model 4**

|  | **HR [IC 95%]** | ***p*** |
| --- | --- | --- |
| Age | 0.95 [0.85–1.05] | 0.30 |
| Female sex | 9.8 [1.49–196.8] | **0.04** |
| Tumor size (cm) |  |  |
| ≤ 1 cm | Ref | - |
| 1.1 cm - 2 cm | 0.37 [0.02–3.53] | 0.43 |
| 2.1 cm - 3 cm | 0.24 [0.01–2.62] | 0.28 |
| ≥ 3.1 cm | 0.49 [0.01–12.12] | 0.67 |
| Degree of emphysema |  |  |
| Light-moderate | Ref | - |
| Severe | 0.49 [0.09–2.6] | 0.39 |
| DLCO (%) |  |  |
| Low (≤ 75) | Ref | - |
| High (> 75) | 4.09 [0.8–25.95] | 0.10 |

**AIC = 66**

## **Model 5**

|  | **HR [IC 95%]** | ***p*** |
| --- | --- | --- |
| Age | 0.94 [0.85–1.03] | 0.19 |
| Female sex | 9.96 [1.59–194.91] | **0.04** |
| Degree of emphysema |  |  |
| Light-moderate | Ref | - |
| Severe | 0.45 [0.1–2.05] | 0.30 |
| DLCO (%) |  |  |
| Low (≤ 75) | Ref | - |
| High (> 75) | 3.77 [0.81–21.32] | 0.10 |

**AIC = 61**

## **Model 6**

|  | **HR [IC 95%]** | ***p*** |
| --- | --- | --- |
| Age | 0.92 [0.84-1] | 0.07 |
| Female sex | 5.71 [1.25–41.64] | **0.04** |
| Degree of emphysema |  |  |
| Light-moderate | Ref | - |
| Severe | 0.2 [0.05–0.75] | **0.02** |

**AIC = 65**

## **Model 7**

|  | **HR [IC 95%]** | ***p*** |
| --- | --- | --- |
| Age | 0.95 [0.85–1.05] | 0.30 |
| Female sex | 5.34 [1.06–42.9] | 0.06 |
| Tumor size (cm) |  |  |
| ≤ 1 cm | Ref | - |
| 1.1 cm - 2 cm | 0.4 [0.02–3.82] | 0.47 |
| 2.1 cm - 3 cm | 0.18 [0.01–1.89] | 0.2 |
| ≥3.1 cm | 0.37 [0.01–8.24] | 0.54 |
| Degree of emphysema |  |  |
| Light-moderate | Ref | - |
| Severe | 0.33 [0.09–1.54] | 0.16 |
| DLCO (%) |  |  |
| Low (≤ 75) | Ref | - |
| High (> 75) | 3.22 [0.8–18.98] | 0.16 |
| FEV1/FVC (%)  Low (≤ 70)  High (> 70) | Ref  1 [0.92–1.08] | 0.98 |

**AIC= 69.25**

## **Model 8**

|  | **HR [IC 95%]** | ***p*** |
| --- | --- | --- |
| Age | 0.94 [0.85–1.03] | 0.17 |
| Female sex | 5.79 [1.3–41.14] | **0.04** |
| FEV1/FVC (%) |  |  |
| Low (≤ 70) | Ref | - |
| High (> 70) | 1.54 [0.38–6.46] | 0.54 |
| DLCO (%) |  |  |
| Low (≤ 75) | Ref | - |
| High (> 75) | 3.75 [0.89–19.72] | 0.08 |

**AIC= 66.737**

AIC, Akaike information criterion; cm, centimeter; DLCO, diffusing capacity for carbon monoxide; FEV1/FVC, forced expiratory volume in one second to forced vital capacity ratio*;* HR, hazard ratio; [IC 95%], confidence interval at 95%; SLN, sentinel lymph node.
